# Crystal structure of ammonium 3′-azido-3′-de­oxy­thymidine-5′-amino­carbonyl­phospho­nate hemi­hydrate: an anti-HIV agent

**DOI:** 10.1107/S1600536814022405

**Published:** 2014-10-24

**Authors:** Maxim V. Jasko, Galina V. Gurskaya, Marina K. Kukhanova, Ivan S. Bushmarinov

**Affiliations:** a Engelhardt Institute of Molecular Biology, Russian Academy of Sciences, 119991, Vavilov St 32, Moscow, Russian Federation; b A.N. Nesmeyanov Institute of Organoelement Compounds, Russian Academy of Sciences, 119991, Vavilov St 28, Moscow, Russian Federation

**Keywords:** crystal structure, anti-HIV agent, de­oxy­thymidine, amino­carbonyl­phospho­nate, ammonium salt

## Abstract

The asymmetric unit of the title compound, NH_4_^+^·C_11_H_14_N_6_O_7_P^−^·0.5H_2_O, contains one 3′-azido-3′-de­oxy­thymidine-5′amino­carbonyl­phospho­nate (ACP–AZT) anion, half of an NH_4_^+^ cation lying on a twofold rotation axis and in another position, occupied with equal probabilities of 0.5, an NH_4_^+^ cation and a water mol­ecule. The amide group of the ACP–AZT anion is disordered (occupancy ratio 0.5:0.5), with one part forming an N—H⋯O (involving C=O⋯H_4_N^+^) hydrogen bond and the other an O—H⋯N (involving C—NH_2_⋯OH_2_) hydrogen bond with the components of the split NH_4_^+^/H_2_O position. The pseudorotation parameters of ACP–AZT set it apart from previously studied AZT and thymidine. In the crystal, the various components are linked by N—H⋯O, O—H⋯O, N—H⋯N, C—H⋯O and C—H⋯N hydrogen bonds, forming a three-dimensional framework.

## Abstract 2

The asymmetric unit of the title compound contains one 3′-azido-3′-de­oxy­thymidine-5′amino­carbonyl­phospho­nate (ACP–AZT) anion, half on an NH_4_^+^ cation lying on a twofold rotation axis and, in another position occupied with equal probabilities of 0.5, an NH_4_^+^ cation and a water mol­ecule.

## Chemical context   

Nucleoside analogues play an important role in clinics as anti­viral drugs. At present, seven nucleoside analogues have been approved by the US FDA for the treatment of HIV-infected patients, the first of which was 3′-azido-3′-de­oxy­thymidine (AZT) (DeClercq, 2010 ▶). Despite progress in the treatment of HIV-infected patients, these drugs possess some drawbacks: AZT lifetime in patients is only one h, requiring frequent dose administration; long-term usage of AZT causes toxic side effects, *viz* anaemia, bone-marrow suppression, neuropathy and emergence of HIV-resistant strains (Stańczak *et al.*, 2006 ▶; Beaumont *et al.*, 2003 ▶). Various forms of nucleosides and nucleotides have been developed in order to reduce the toxic effects of anti-HIV drugs, to increase their oral bioavailability and to improve their pharmacokinetic properties (Kukhanova & Shirokova, 2005 ▶). Out of a large number of potential HIV drugs, only one compound has been approved by the FDA for the treatment of HIV-infected patients, namely, tenofovir disoproxil fumarate (Viread^®^; DeClercq, 2010 ▶), and one prodrug of AZT (5′-hydrogenphospho­nate AZT, Nikavir^®^) has been used in clinical trials in Russia (Ivanova *et al.*, 2010 ▶; Kukhanova & Shirokova, 2005 ▶). In a continuation of the search for compounds with improved medicinal properties, we have synthesized a novel derivative form of AZT, 5′-amino­carbonyl­phospho­nate 3′-azido-3′-de­oxy­thymidine (ACP–AZT). Biological testing of ACP–AZT in cell cultures infected with HIV-1 showed that this compound inhibited virus replication and its toxicity was much lower compared to that of AZT and Nikavir. ACP–AZT displayed improved pharmacokinetic characteristics com­pared to AZT (Khandazhinskaya *et al.*, 2009 ▶; Kukhanova, 2012 ▶; Shirokova *et al.*, 2006 ▶). Accumulation of ACP–AZT in animal blood was slower than the accumulation of AZT, leading to a decrease in the toxic side effects displayed by AZT. The half-life of ACP–AZT in animal blood is three to four times longer than that of AZT, making it a perspective candidate as an anti-HIV drug for clinical usage. At present, the title compound is undergoing clinical trials as a potential anti-HIV drug.
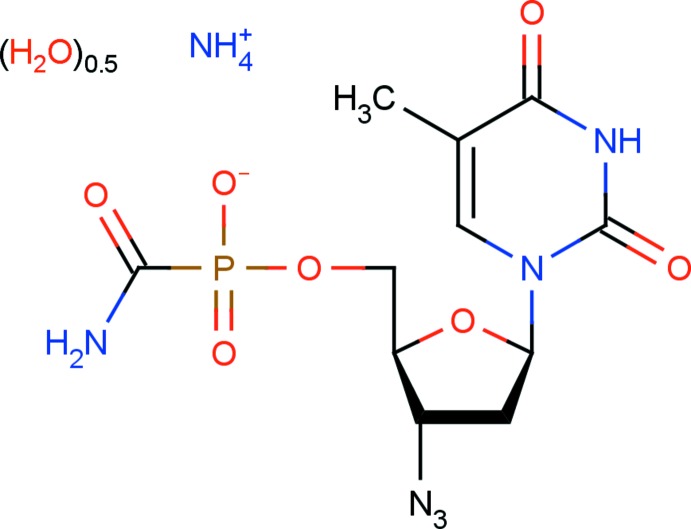


## Structural commentary   

**Figure 1:**
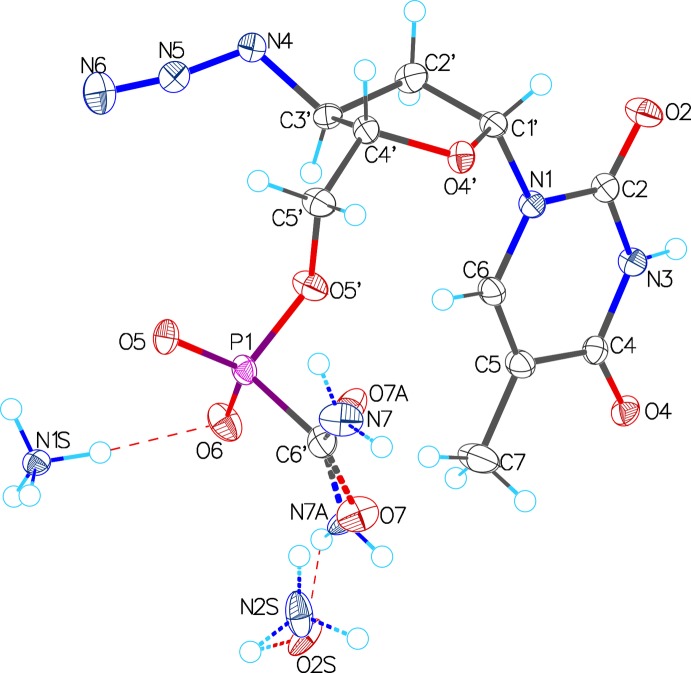
A view of the mol­ecular structure of the title salt, showing the atom numbering. Displacement ellipsoids are drawn at the 50% probability level. The ammonium cation, N1*S*, lies on a twofold rotation axis.

The mol­ecular structure of the title compound, ACP–AZT, is illustrated in Fig. 1 ▶. The comparative analysis of the crystal structure conformation of the title ACP–AZT mol­ecule with the conformation of AZT and natural thymidine mol­ecules (Young *et al.*, 1969 ▶) is discussed below. The main differences are observed in the carbohydrate fragments of the mol­ecules. In terms of pseudorotation (IUPAC–IUB, 1983 ▶), the conformation of the furan­ose ring in the ACP–AZT mol­ecule is described by the phase angle of pseudorotation, *P* = 25.2°, and the degree of pucker, Ψ_m_ = 35.0°. These results correspond to a C3′-*endo*-C4′-*exo* (^3^T_4_) conformation of the sugar cycle. Atoms C3′ and C4′ deviate from the plane of atoms C1′/O4′/C2′ by 0.458 and −0.101 Å, respectively. Unlike the AZT mol­ecules and the mol­ecule of thymidine, which exhibit a C3′-*exo-* class of pucker, the ACP–AZT mol­ecule exhibits a C3′-*endo* pucker. The orientation of the thymine base relative to the de­oxy­ribose ring in the ACP–AZT mol­ecule is *anti*, similar to that in natural thymidine and AZT, the glycosyl torsion angle χ_ACP–AZT_(O4′—C1′—N1—C2) = −147.75 (16)°. The geometric parameters of the azido residue and the orientation relative to the de­oxy­ribose ring in ACP–AZT and AZT coincide within experimental error.

## Supra­molecular features   

**Figure 2:**
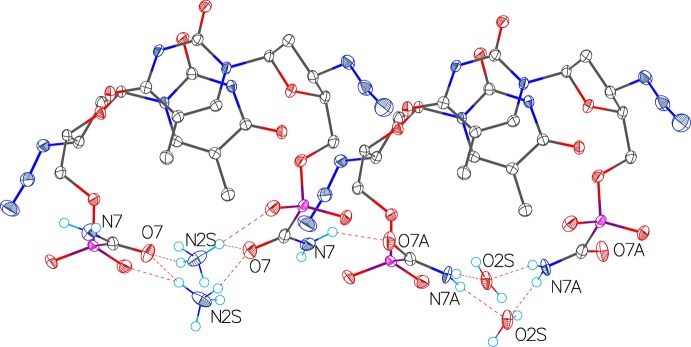
The hydrogen bonds involving the disordered water and ammonia mol­ecules in the crystal packing of ACP–AZT (see Table 1 ▶ for details). A fragment of the hypothetically ordered ‘supercell’ is shown.

The C(O)NH_2_ group of ACP–AZT is disordered, one part forming a C=O⋯H_4_N^+^ hydrogen bond and the other a C—NH_2_⋯OH_2_ hydrogen bond with the components of the NH_4_^+^/H_2_O position (Table 1 ▶ and Fig. 2 ▶). In the crystal, the various components are linked by N—H⋯O, O—H⋯O, N—H⋯N, C—H⋯O and C—H⋯N hydrogen bonds (Table 1 ▶), forming a three-dimensional framework. The structure can be described by an ordered supercell doubled in the *c* direction (Fig. 2 ▶); however, this was not observed in the diffraction experiment.

## Database survey   

Earlier, in 1986, we studied the crystal and mol­ecular structures of AZT and then some other HIV replication inhibitors by X-ray analysis (Gurskaya *et al.*, 1986 ▶, 1990 ▶, 1991 ▶, 1992 ▶). AZT structures obtained later by four other laboratories were similar to our structure (Camerman *et al.*, 1987 ▶; Birnbaum *et al.*, 1987 ▶; Parthasarathy *et al.*, 1988 ▶; Van Roey *et al.*, 1988 ▶).

## Synthesis and crystallization   

The title compound was synthesized as described earlier (Shirokova *et al.*, 2004 ▶). The crystals for X-ray analysis were selected from a highly dispersed (fine crystals) batch of ACP–AZT prepared for clinical usage.

## Refinement   

Crystal data, data collection and structure refinement details are summarized in Table 2 ▶. The C-bound H atoms were included in calculated positions and treated as riding, with C—H = 0.95–1.00 Å and *U*_iso_(H) = 1.5*U*_eq_(C) for methyl H atoms and 1.2*U*_eq_(C) for other H atoms. The other distance restraints and SIMU parameters are given below: DFIX 1.234 0.005 O7*A* C6′ O7 C6′; DFIX 0.9 N7 H7a N7 H7b; DFIX 0.95 N2*S* H2Sc N2*S* H2Sd N2*S* H2Sa N2*S* H2Sb O2*S* H2Sb O2*S* H2Sa; DFIX 1.325 0.005 N7 C6′ N7*A* C6′; DFIX 0.9 N7*A* H7Aa N7*A* H7Ab; SIMU 0.01 0.005 1.7 N2*S* O2*S*; SIMU 0.01 0.005 1.7 N7*A* O7 O7*A* N7. The split NH_4_^+^/H_2_O position was refined with an occupancy of 0.5 for each atom.

## Supplementary Material

Crystal structure: contains datablock(s) global, I. DOI: 10.1107/S1600536814022405/su2792sup1.cif

Structure factors: contains datablock(s) I. DOI: 10.1107/S1600536814022405/su2792Isup2.hkl

CCDC reference: 1028847

Additional supporting information:  crystallographic information; 3D view; checkCIF report

## Figures and Tables

**Table 1:** Hydrogen-bond geometry (, )

*D*H*A*	*D*H	H*A*	*D* *A*	*D*H*A*
N1*S*H1*SA*O4^i^	0.85(2)	2.01(2)	2.8565(19)	173(2)
N1*S*H1*SB*O6	0.94(3)	1.86(3)	2.780(2)	168(2)
N3H3O5^ii^	0.90(3)	1.90(3)	2.781(2)	167(3)
O2*S*H2*SA*O5^iii^	0.87(2)	2.00(2)	2.868(11)	176(3)
N2*S*H2*SA*O5^iii^	0.93(2)	2.00(2)	2.857(11)	154(3)
N2*S*H2*SC*O6	0.94(3)	2.21(4)	3.013(12)	143(5)
N2*S*H2*SC*O7	0.94(3)	2.20(5)	2.901(16)	130(5)
O2*S*H2*SB*O2^iv^	0.93(2)	1.91(2)	2.822(11)	166(3)
N2*S*H2*SB*O2^iv^	0.95(2)	1.91(2)	2.818(12)	159(3)
N2*S*H2*SD*O7^v^	0.95(3)	1.99(3)	2.902(16)	162(5)
N7H7*A*N7^vi^	0.91(3)	1.93(5)	2.67(3)	136(5)
N7H7*B*N2*S* ^vii^	0.92(3)	2.66(6)	3.265(17)	124(5)
N7*A*H7*AA*O2*S* ^v^	0.90(3)	2.00(3)	2.887(18)	167(6)
N7*A*H7*AB*O2*S*	0.91(3)	2.03(3)	2.856(15)	150(4)
C1H1O6^viii^	0.90(3)	2.53(2)	3.100(2)	122.3(19)
C3H3N4^iv^	0.92(2)	2.65(2)	3.274(3)	125.1(19)
C4H4O4^ix^	1.00	2.51	3.257(2)	131
C6H6O5	0.95	2.47	3.402(2)	168

Symmetry codes: (i) 
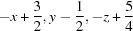
; (ii) 
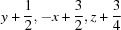
; (iii) 
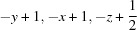
; (iv) 
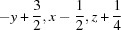
; (v) 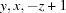; (vi) 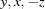; (vii) 
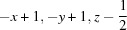
; (viii) 
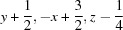
; (ix) 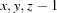.

**Table 2:** Experimental details

Crystal data	
Chemical formula	NH_4_^+^C_11_H_14_N_6_O_7_P0.5H_2_O
*M*_r_	400.30
Crystal system, space group	Tetragonal, *P*4_1_2_1_2
Temperature (K)	100
*a*, *c* ()	18.5564(6), 10.1139(4)
*V* (^3^)	3482.6(3)
*Z*	8
Radiation type	Mo *K*
(mm^1^)	0.21
Crystal size (mm)	0.21 0.20 0.20

Data collection	
Diffractometer	Bruker APEXII CCD
Absorption correction	Multi-scan (*SADABS*; Bruker, 2009 ▶)
*T*_min_, *T*_max_	0.670, 0.746
No. of measured, independent and observed [*I* > 2(*I*)] reflections	47562, 5322, 4822
*R*_int_	0.047
(sin /)_max_ (^1^)	0.714

Refinement	
*R*[*F* ^2^ > 2(*F* ^2^)], *wR*(*F* ^2^), *S*	0.032, 0.079, 1.09
No. of reflections	5322
No. of parameters	315
No. of restraints	32
H-atom treatment	H atoms treated by a mixture of independent and constrained refinement
_max_, _min_ (e ^3^)	0.34, 0.29
Absolute structure	Flack x determined using 1919 quotients [(*I* ^+^)(*I* )]/[(*I* ^+^)+(*I* )] (Parsons *et al.*, 2013 ▶)
Absolute structure parameter	0.00(3)

Computer programs: *APEX2* and *SAINT* (Bruker, 2009 ▶), *SHELXS2014* and *SHELXL2014* (Sheldrick, 2008 ▶) and *OLEX2* (Dolomanov *et al.*, 2009 ▶).

## supplementary crystallographic information

### Crystal data

**Table:**

NH_4_^+^·C_11_H_14_N_6_O_7_P^−^·0.5H_2_O	*D*_x_ = 1.527 Mg m^−^^3^
*M**_r_* = 400.30	Mo *K*α radiation, λ = 0.71073 Å
Tetragonal, *P*4_1_2_1_2	Cell parameters from 9998 reflections
*a* = 18.5564 (6) Å	θ = 2.3–30.3°
*c* = 10.1139 (4) Å	µ = 0.21 mm^−^^1^
*V* = 3482.6 (3) Å^3^	*T* = 100 K
*Z* = 8	Prism, colourless
*F*(000) = 1672	0.21 × 0.20 × 0.20 mm

### Data collection

**Table:**

Bruker APEXII CCD diffractometer	5322 independent reflections
Radiation source: sealed tube	4822 reflections with *I* > 2σ(*I*)
Graphite monochromator	*R*_int_ = 0.047
Detector resolution: 8 pixels mm^-1^	θ_max_ = 30.5°, θ_min_ = 2.2°
φ and ω scans	*h* = −26→26
Absorption correction: multi-scan (*SADABS*; Bruker, 2009)	*k* = −26→26
*T*_min_ = 0.670, *T*_max_ = 0.746	*l* = −14→14
47562 measured reflections	

### Refinement

**Table:**

Refinement on *F*^2^	Secondary atom site location: difference Fourier map
Least-squares matrix: full	Hydrogen site location: mixed
*R*[*F*^2^ > 2σ(*F*^2^)] = 0.032	H atoms treated by a mixture of independent and constrained refinement
*wR*(*F*^2^) = 0.079	*w* = 1/[σ^2^(*F*_o_^2^) + (0.0415*P*)^2^ + 0.5648*P*] where *P* = (*F*_o_^2^ + 2*F*_c_^2^)/3
*S* = 1.09	(Δ/σ)_max_ = 0.001
5322 reflections	Δρ_max_ = 0.34 e Å^−^^3^
315 parameters	Δρ_min_ = −0.29 e Å^−^^3^
32 restraints	Absolute structure: Flack x determined using 1919 quotients [(*I*^+^)-(*I*^-^)]/[(*I*^+^)+(*I*^-^)] (Parsons *et al.*, 2013)
Primary atom site location: structure-invariant direct methods	Absolute structure parameter: 0.00 (3)

### Special details

**Table:**

Geometry. All e.s.d.'s (except the e.s.d. in the dihedral angle between two l.s. planes) are estimated using the full covariance matrix. The cell e.s.d.'s are taken into account individually in the estimation of e.s.d.'s in distances, angles and torsion angles; correlations between e.s.d.'s in cell parameters are only used when they are defined by crystal symmetry. An approximate (isotropic) treatment of cell e.s.d.'s is used for estimating e.s.d.'s involving l.s. planes.

### Fractional atomic coordinates and isotropic or equivalent isotropic displacement parameters (Å^2^)

**Table:**

	*x*	*y*	*z*	*U*_iso_*/*U*_eq_	Occ. (<1)
P1	0.68921 (3)	0.49006 (3)	0.20527 (5)	0.01645 (10)	
O2	0.92567 (8)	0.79475 (9)	0.50029 (14)	0.0247 (3)	
O2S	0.5859 (5)	0.4368 (6)	0.5786 (9)	0.0241 (17)	0.5
O4	0.79798 (8)	0.68215 (7)	0.82247 (13)	0.0194 (3)	
O4'	0.81753 (7)	0.69188 (7)	0.21627 (13)	0.0172 (3)	
O5	0.68747 (8)	0.45502 (8)	0.07236 (14)	0.0229 (3)	
O5'	0.74688 (8)	0.55407 (8)	0.20885 (14)	0.0234 (3)	
O6	0.70278 (9)	0.44576 (8)	0.32578 (14)	0.0257 (3)	
O7	0.5715 (9)	0.5389 (8)	0.3380 (10)	0.026 (2)	0.5
O7A	0.5847 (6)	0.5815 (6)	0.1427 (10)	0.0303 (19)	0.5
N1	0.85030 (9)	0.70295 (9)	0.43868 (15)	0.0156 (3)	
N1S	0.69819 (9)	0.30181 (9)	0.2500	0.0156 (4)	
H1SA	0.7013 (13)	0.2684 (13)	0.308 (2)	0.018 (6)*	
H1SB	0.7014 (14)	0.3480 (14)	0.287 (3)	0.025 (6)*	
H3	0.8853 (15)	0.7633 (15)	0.719 (3)	0.029*	
H2SA	0.5720 (15)	0.3982 (11)	0.536 (3)	0.029*	
H2SC	0.619 (3)	0.461 (3)	0.478 (4)	0.029*	0.5
H2SB	0.6270 (12)	0.4260 (15)	0.628 (2)	0.029*	
H2SD	0.570 (3)	0.477 (3)	0.590 (5)	0.029*	0.5
N2S	0.5985 (7)	0.4395 (7)	0.5538 (11)	0.028 (2)	0.5
N3	0.86108 (9)	0.73708 (9)	0.65883 (15)	0.0163 (3)	
N4	0.94590 (10)	0.55785 (9)	0.10114 (18)	0.0229 (4)	
N5	0.92571 (10)	0.49900 (10)	0.05676 (19)	0.0266 (4)	
N6	0.91507 (14)	0.44520 (13)	0.0082 (3)	0.0464 (6)	
N7	0.5716 (7)	0.5749 (8)	0.1320 (14)	0.0259 (17)	0.5
H7A	0.597 (3)	0.578 (3)	0.055 (4)	0.037 (16)*	0.5
H7B	0.531 (2)	0.604 (3)	0.133 (6)	0.038 (16)*	0.5
N7A	0.5747 (10)	0.5256 (10)	0.3488 (11)	0.0199 (17)	0.5
H7AA	0.532 (2)	0.549 (3)	0.361 (6)	0.033 (17)*	0.5
H7AB	0.593 (2)	0.494 (2)	0.407 (4)	0.011 (11)*	0.5
C1'	0.87645 (10)	0.70731 (10)	0.30064 (18)	0.0166 (3)	
H1'	0.8922 (13)	0.7528 (14)	0.290 (2)	0.016 (6)*	
C2	0.88180 (10)	0.74798 (10)	0.52988 (18)	0.0164 (3)	
C2'	0.93586 (11)	0.65280 (11)	0.26815 (19)	0.0206 (4)	
H2'A	0.9735	0.6747	0.2116	0.025*	
H2'B	0.9586	0.6341	0.3498	0.025*	
C3'	0.89591 (10)	0.59331 (10)	0.19471 (19)	0.0168 (3)	
H3'	0.8762 (13)	0.5614 (14)	0.255 (2)	0.018 (6)*	
C4	0.81153 (10)	0.68706 (10)	0.70369 (17)	0.0154 (3)	
C4'	0.83606 (10)	0.63509 (10)	0.12478 (17)	0.0163 (3)	
H4'	0.8557	0.6569	0.0419	0.020*	
C5	0.77850 (11)	0.64287 (11)	0.60195 (19)	0.0204 (4)	
C5'	0.76867 (12)	0.59301 (11)	0.09211 (19)	0.0211 (4)	
H5'A	0.7298	0.6262	0.0640	0.025*	
H5'B	0.7784	0.5589	0.0189	0.025*	
C6	0.79969 (11)	0.65210 (10)	0.47609 (19)	0.0198 (4)	
H6	0.7790	0.6224	0.4097	0.024*	
C6'	0.60339 (12)	0.53904 (11)	0.23020 (18)	0.0207 (4)	
C7	0.72211 (16)	0.58965 (16)	0.6417 (2)	0.0416 (7)	
H7C	0.7422	0.5562	0.7070	0.062*	
H7D	0.7061	0.5627	0.5638	0.062*	
H7E	0.6810	0.6152	0.6805	0.062*	

### Atomic displacement parameters (Å^2^)

**Table:**

	*U*^11^	*U*^22^	*U*^33^	*U*^12^	*U*^13^	*U*^23^
P1	0.0215 (2)	0.0144 (2)	0.01346 (19)	−0.00265 (18)	−0.00230 (17)	−0.00192 (16)
O2	0.0285 (8)	0.0300 (8)	0.0157 (6)	−0.0136 (6)	−0.0020 (6)	0.0018 (6)
O2S	0.024 (3)	0.033 (3)	0.015 (2)	0.017 (2)	0.0025 (18)	−0.0010 (17)
O4	0.0261 (7)	0.0193 (6)	0.0127 (6)	0.0011 (5)	0.0019 (5)	−0.0005 (5)
O4'	0.0213 (6)	0.0177 (6)	0.0125 (5)	0.0009 (5)	−0.0039 (5)	−0.0014 (5)
O5	0.0287 (8)	0.0212 (7)	0.0189 (6)	−0.0021 (6)	0.0005 (6)	−0.0088 (5)
O5'	0.0289 (8)	0.0269 (7)	0.0143 (6)	−0.0122 (6)	−0.0027 (6)	0.0011 (6)
O6	0.0348 (8)	0.0200 (7)	0.0223 (7)	−0.0033 (6)	−0.0079 (6)	0.0049 (5)
O7	0.026 (2)	0.033 (5)	0.018 (2)	0.008 (3)	−0.0044 (19)	0.0120 (19)
O7A	0.041 (5)	0.031 (3)	0.019 (2)	0.012 (3)	0.007 (3)	0.000 (2)
N1	0.0213 (7)	0.0161 (7)	0.0095 (6)	−0.0030 (6)	−0.0009 (6)	−0.0001 (5)
N1S	0.0175 (6)	0.0175 (6)	0.0119 (9)	−0.0014 (8)	0.0005 (6)	0.0005 (6)
N2S	0.025 (4)	0.018 (2)	0.042 (6)	0.006 (2)	−0.015 (4)	−0.008 (3)
N3	0.0190 (8)	0.0190 (8)	0.0110 (6)	−0.0029 (6)	−0.0028 (6)	−0.0001 (6)
N4	0.0264 (9)	0.0184 (8)	0.0239 (8)	−0.0024 (7)	0.0071 (7)	−0.0026 (7)
N5	0.0293 (9)	0.0229 (9)	0.0276 (9)	−0.0021 (7)	0.0081 (8)	−0.0030 (7)
N6	0.0528 (14)	0.0334 (11)	0.0529 (14)	−0.0122 (10)	0.0144 (12)	−0.0194 (11)
N7	0.016 (3)	0.039 (3)	0.023 (3)	−0.002 (2)	−0.0084 (18)	0.008 (3)
N7A	0.022 (3)	0.028 (5)	0.009 (2)	0.009 (3)	0.005 (2)	0.006 (2)
C1'	0.0214 (8)	0.0183 (8)	0.0101 (7)	−0.0043 (7)	0.0002 (6)	−0.0005 (6)
C2	0.0182 (8)	0.0173 (8)	0.0138 (8)	−0.0007 (7)	−0.0025 (7)	0.0002 (7)
C2'	0.0184 (9)	0.0276 (10)	0.0158 (8)	−0.0021 (7)	−0.0014 (7)	−0.0026 (7)
C3'	0.0189 (8)	0.0182 (8)	0.0132 (7)	−0.0005 (7)	0.0022 (7)	0.0014 (7)
C4	0.0171 (8)	0.0142 (7)	0.0148 (7)	0.0017 (7)	−0.0009 (7)	0.0002 (6)
C4'	0.0227 (9)	0.0156 (8)	0.0107 (7)	−0.0021 (7)	−0.0011 (7)	−0.0001 (6)
C5	0.0241 (10)	0.0205 (9)	0.0165 (8)	−0.0068 (8)	0.0012 (7)	−0.0017 (7)
C5'	0.0260 (10)	0.0251 (10)	0.0123 (7)	−0.0080 (8)	−0.0019 (7)	0.0012 (7)
C6	0.0226 (9)	0.0186 (9)	0.0182 (9)	−0.0066 (7)	0.0015 (7)	−0.0037 (7)
C6'	0.0277 (10)	0.0203 (9)	0.0140 (8)	−0.0016 (7)	−0.0016 (7)	−0.0009 (7)
C7	0.0536 (16)	0.0472 (15)	0.0242 (11)	−0.0338 (13)	0.0121 (11)	−0.0092 (11)

### Geometric parameters (Å, º)

**Table:**

P1—O5	1.4936 (14)	N4—C3'	1.480 (3)
P1—O5'	1.5991 (14)	N5—N6	1.130 (3)
P1—O6	1.4916 (15)	N7—H7A	0.91 (3)
P1—C6'	1.851 (2)	N7—H7B	0.92 (3)
O2—C2	1.227 (2)	N7—C6'	1.333 (6)
O2S—H2SA	0.874 (19)	N7A—H7AA	0.90 (3)
O2S—H2SB	0.93 (2)	N7A—H7AB	0.91 (3)
O4—C4	1.231 (2)	N7A—C6'	1.336 (5)
O4'—C1'	1.416 (2)	C1'—H1'	0.90 (3)
O4'—C4'	1.444 (2)	C1'—C2'	1.532 (3)
O5'—C5'	1.442 (2)	C2'—H2'A	0.9900
O7—C6'	1.241 (6)	C2'—H2'B	0.9900
O7A—C6'	1.234 (5)	C2'—C3'	1.523 (3)
N1—C1'	1.480 (2)	C3'—H3'	0.92 (2)
N1—C2	1.375 (2)	C3'—C4'	1.528 (3)
N1—C6	1.384 (2)	C4—C5	1.452 (3)
N1S—H1SA	0.85 (2)	C4'—H4'	1.0000
N1S—H1SB	0.94 (3)	C4'—C5'	1.511 (3)
N2S—H2SA	0.93 (2)	C5—C6	1.343 (3)
N2S—H2SC	0.94 (3)	C5—C7	1.494 (3)
N2S—H2SB	0.95 (2)	C5'—H5'A	0.9900
N2S—H2SD	0.95 (3)	C5'—H5'B	0.9900
N3—H3	0.90 (3)	C6—H6	0.9500
N3—C2	1.375 (2)	C7—H7C	0.9800
N3—C4	1.383 (2)	C7—H7D	0.9800
N4—N5	1.239 (2)	C7—H7E	0.9800
			
O5—P1—O5'	110.99 (8)	C3'—C2'—C1'	103.47 (15)
O5—P1—C6'	108.53 (9)	C3'—C2'—H2'A	111.1
O5'—P1—C6'	102.01 (9)	C3'—C2'—H2'B	111.1
O6—P1—O5	119.94 (9)	N4—C3'—C2'	109.21 (16)
O6—P1—O5'	106.13 (8)	N4—C3'—H3'	112.6 (15)
O6—P1—C6'	107.73 (9)	N4—C3'—C4'	112.67 (15)
H2SA—O2S—H2SB	109 (3)	C2'—C3'—H3'	109.6 (15)
C1'—O4'—C4'	110.47 (14)	C2'—C3'—C4'	102.23 (15)
C5'—O5'—P1	122.76 (12)	C4'—C3'—H3'	109.9 (15)
C2—N1—C1'	117.39 (15)	O4—C4—N3	120.38 (17)
C2—N1—C6	121.29 (16)	O4—C4—C5	124.32 (17)
C6—N1—C1'	121.17 (15)	N3—C4—C5	115.30 (16)
H1SA—N1S—H1SB	113 (2)	O4'—C4'—C3'	104.29 (14)
H2SA—N2S—H2SC	113 (4)	O4'—C4'—H4'	109.2
H2SA—N2S—H2SB	103 (3)	O4'—C4'—C5'	108.68 (16)
H2SA—N2S—H2SD	113 (4)	C3'—C4'—H4'	109.2
H2SC—N2S—H2SB	122 (4)	C5'—C4'—C3'	116.14 (16)
H2SC—N2S—H2SD	103 (5)	C5'—C4'—H4'	109.2
H2SB—N2S—H2SD	101 (4)	C4—C5—C7	118.58 (17)
C2—N3—H3	114.9 (18)	C6—C5—C4	118.44 (18)
C2—N3—C4	126.57 (16)	C6—C5—C7	122.99 (18)
C4—N3—H3	118.3 (18)	O5'—C5'—C4'	108.18 (15)
N5—N4—C3'	115.73 (17)	O5'—C5'—H5'A	110.1
N6—N5—N4	171.6 (2)	O5'—C5'—H5'B	110.1
H7A—N7—H7B	113 (5)	C4'—C5'—H5'A	110.1
C6'—N7—H7A	116 (4)	C4'—C5'—H5'B	110.1
C6'—N7—H7B	130 (4)	H5'A—C5'—H5'B	108.4
H7AA—N7A—H7AB	124 (5)	N1—C6—H6	118.5
C6'—N7A—H7AA	112 (4)	C5—C6—N1	123.00 (17)
C6'—N7A—H7AB	124 (3)	C5—C6—H6	118.5
O4'—C1'—N1	107.70 (14)	O7—C6'—P1	121.9 (6)
O4'—C1'—H1'	111.8 (15)	O7—C6'—N7	116.4 (11)
O4'—C1'—C2'	107.03 (15)	O7A—C6'—P1	117.2 (6)
N1—C1'—H1'	105.5 (16)	O7A—C6'—N7A	130.6 (9)
N1—C1'—C2'	113.71 (15)	N7—C6'—P1	121.6 (8)
C2'—C1'—H1'	111.1 (15)	N7A—C6'—P1	111.9 (7)
O2—C2—N1	123.25 (17)	C5—C7—H7C	109.5
O2—C2—N3	121.39 (17)	C5—C7—H7D	109.5
N3—C2—N1	115.35 (16)	C5—C7—H7E	109.5
C1'—C2'—H2'A	111.1	H7C—C7—H7D	109.5
C1'—C2'—H2'B	111.1	H7C—C7—H7E	109.5
H2'A—C2'—H2'B	109.0	H7D—C7—H7E	109.5
			
P1—O5'—C5'—C4'	−165.61 (14)	C1'—O4'—C4'—C3'	24.84 (18)
O4—C4—C5—C6	−177.9 (2)	C1'—O4'—C4'—C5'	149.32 (15)
O4—C4—C5—C7	2.6 (3)	C1'—N1—C2—O2	6.0 (3)
O4'—C1'—C2'—C3'	−18.00 (19)	C1'—N1—C2—N3	−173.72 (16)
O4'—C4'—C5'—O5'	−68.8 (2)	C1'—N1—C6—C5	174.96 (19)
O5—P1—O5'—C5'	25.85 (19)	C1'—C2'—C3'—N4	151.29 (15)
O5—P1—C6'—O7	139.4 (11)	C1'—C2'—C3'—C4'	31.75 (18)
O5—P1—C6'—O7A	−52.1 (6)	C2—N1—C1'—O4'	−147.75 (16)
O5—P1—C6'—N7	−38.8 (8)	C2—N1—C1'—C2'	93.8 (2)
O5—P1—C6'—N7A	132.9 (11)	C2—N1—C6—C5	−0.5 (3)
O5'—P1—C6'—O7	−103.3 (11)	C2—N3—C4—O4	179.38 (18)
O5'—P1—C6'—O7A	65.2 (6)	C2—N3—C4—C5	−0.6 (3)
O5'—P1—C6'—N7	78.4 (8)	C2'—C3'—C4'—O4'	−34.79 (18)
O5'—P1—C6'—N7A	−109.8 (11)	C2'—C3'—C4'—C5'	−154.33 (16)
O6—P1—O5'—C5'	157.72 (16)	C3'—C4'—C5'—O5'	48.3 (2)
O6—P1—C6'—O7	8.1 (11)	C4—N3—C2—O2	178.93 (18)
O6—P1—C6'—O7A	176.6 (6)	C4—N3—C2—N1	−1.3 (3)
O6—P1—C6'—N7	−170.1 (8)	C4—C5—C6—N1	−1.6 (3)
O6—P1—C6'—N7A	1.6 (11)	C4'—O4'—C1'—N1	−126.92 (15)
N1—C1'—C2'—C3'	100.81 (17)	C4'—O4'—C1'—C2'	−4.29 (19)
N3—C4—C5—C6	2.0 (3)	C6—N1—C1'—O4'	36.6 (2)
N3—C4—C5—C7	−177.5 (2)	C6—N1—C1'—C2'	−81.8 (2)
N4—C3'—C4'—O4'	−151.88 (15)	C6—N1—C2—O2	−178.36 (19)
N4—C3'—C4'—C5'	88.6 (2)	C6—N1—C2—N3	1.9 (3)
N5—N4—C3'—C2'	164.58 (18)	C6'—P1—O5'—C5'	−89.59 (17)
N5—N4—C3'—C4'	−82.6 (2)	C7—C5—C6—N1	177.9 (2)

### Hydrogen-bond geometry (Å, º)

**Table:**

*D*—H···*A*	*D*—H	H···*A*	*D*···*A*	*D*—H···*A*
N1*S*—H1*SA*···O4^i^	0.85 (2)	2.01 (2)	2.8565 (19)	173 (2)
N1*S*—H1*SB*···O6	0.94 (3)	1.86 (3)	2.780 (2)	168 (2)
N3—H3···O5^ii^	0.90 (3)	1.90 (3)	2.781 (2)	167 (3)
O2*S*—H2*SA*···O5^iii^	0.87 (2)	2.00 (2)	2.868 (11)	176 (3)
N2*S*—H2*SA*···O5^iii^	0.93 (2)	2.00 (2)	2.857 (11)	154 (3)
N2*S*—H2*SC*···O6	0.94 (3)	2.21 (4)	3.013 (12)	143 (5)
N2*S*—H2*SC*···O7	0.94 (3)	2.20 (5)	2.901 (16)	130 (5)
O2*S*—H2*SB*···O2^iv^	0.93 (2)	1.91 (2)	2.822 (11)	166 (3)
N2*S*—H2*SB*···O2^iv^	0.95 (2)	1.91 (2)	2.818 (12)	159 (3)
N2*S*—H2*SD*···O7^v^	0.95 (3)	1.99 (3)	2.902 (16)	162 (5)
N7—H7*A*···N7^vi^	0.91 (3)	1.93 (5)	2.67 (3)	136 (5)
N7—H7*B*···N2*S*^vii^	0.92 (3)	2.66 (6)	3.265 (17)	124 (5)
N7*A*—H7*AA*···O2*S*^v^	0.90 (3)	2.00 (3)	2.887 (18)	167 (6)
N7*A*—H7*AB*···O2*S*	0.91 (3)	2.03 (3)	2.856 (15)	150 (4)
C1′—H1′···O6^viii^	0.90 (3)	2.53 (2)	3.100 (2)	122.3 (19)
C3′—H3′···N4^iv^	0.92 (2)	2.65 (2)	3.274 (3)	125.1 (19)
C4′—H4′···O4^ix^	1.00	2.51	3.257 (2)	131
C6—H6···O5′	0.95	2.47	3.402 (2)	168

Symmetry codes: (i) −*x*+3/2, *y*−1/2, −*z*+5/4; (ii) *y*+1/2, −*x*+3/2, *z*+3/4; (iii) −*y*+1, −*x*+1, −*z*+1/2; (iv) −*y*+3/2, *x*−1/2, *z*+1/4; (v) *y*, *x*, −*z*+1; (vi) *y*, *x*, −*z*; (vii) −*x*+1, −*y*+1, *z*−1/2; (viii) *y*+1/2, −*x*+3/2, *z*−1/4; (ix) *x*, *y*, *z*−1.
